# Knowledge, Attitudes and Practical Experience With CAD/CAM Technology Among Dental Students in Croatia: A Cross‐Sectional Study

**DOI:** 10.1111/eje.70126

**Published:** 2026-03-03

**Authors:** Sanja Peršić Kiršić, Petra Rajčić, Mia Vujević, Žarko Udiljak

**Affiliations:** ^1^ Department of Prosthodontics School of Dental Medicine University of Zagreb Zagreb Croatia; ^2^ Private Dental Clinics Zagreb Croatia; ^3^ Private Dental Clinics Split Croatia; ^4^ Department of Prosthodontics Faculty of Dental Medicine and Health, Josip Juraj Strossmayer University of Osijek Osijek Croatia

**Keywords:** CAD/CAM technology, curriculum development, dental education, dental students, digital dentistry, knowledge and attitudes

## Abstract

**Introduction:**

CAD/CAM (Computer‐Aided Design/Computer‐Aided Manufacturing) technology has become integral to modern dental practice, requiring structured integration into dental education. However, little is known about how well dental students in Croatia understand and engage with these technologies. This study aimed to quantitatively assess the level of knowledge, attitudes and practical experience with CAD/CAM among dental students in Osijek, and to explore the influence of academic year, gender and family background in dentistry on these dimensions.

**Materials and Methods:**

A cross‐sectional survey was conducted at the Faculty of Dental Medicine and Health in Osijek, including 102 students from the 3rd to 6th year., with 44 (44.88%) in preclinical and 58 (55.12%) in clinical training. A 21‐item questionnaire assessed theoretical knowledge, practical exposure and attitudes. Data were analysed using descriptive statistics, ANOVA, *t*‐tests and chi‐square tests (*p* < 0.05).

**Results:**

While 88.24% of students had heard of CAD/CAM, only 45.10% had practical experience and just 11.76% had attended extracurricular CAD/CAM training. Nonetheless, 90.20% believed CAD/CAM should be more thoroughly taught, and 85.29% expressed interest in further education. Clinical‐year students and those with family in dentistry showed significantly higher knowledge and more positive attitudes (*p* < 0.001), whereas no significant gender differences were observed (*p* = 0.222).

**Conclusion:**

The findings indicate a gap between theoretical familiarity and practical competence among Croatian dental students regarding CAD/CAM technology. To address this, we recommend the development of a structured undergraduate CAD/CAM module that includes theoretical instruction, digital simulation and supervised clinical training across all study years. Such curriculum development is essential to prepare future dentists for the demands of digital clinical practice.

## 1 Introduction

In recent decades, rapid advancements in technology—particularly in computing and information systems—have profoundly impacted nearly all aspects of daily life, including dental education and professional development in the field of dental medicine. The integration of digital technologies has significantly transformed traditional clinical workflows, especially within prosthodontics. One of the most influential advancements in this area is the CAD/CAM (Computer‐Aided Design/Computer‐Aided Manufacturing) system, which enables the digital design and fabrication of dental restorations. Since its introduction in the late 20th century, CAD/CAM technology has evolved continuously and is now considered an essential component of contemporary dental practice [1, 2, 3].

CAD/CAM systems streamline the production of both fixed and removable prostheses by replacing many manual and time‐consuming procedures with precise digital processes. This not only improves the accuracy, efficiency and reproducibility of prosthetic restorations but also reduces treatment time and enhances patient comfort. The digital workflow typically involves intraoral scanning, virtual design using CAD software and fabrication via computer‐controlled milling machines or 3D printers [3, 4, 5]. In prosthodontics, CAD/CAM technology enables the production of crowns, bridges, veneers, inlays, onlays and complete dentures with a high degree of customization and precision [6, 7, 8].

Given these advantages, there is a growing need to integrate digital technologies, including CAD/CAM systems, into undergraduate dental education. Early exposure to such innovations helps students develop the technical competence and digital literacy required for modern clinical practice. Furthermore, incorporating digital tools into the curriculum early on may foster more favourable attitudes among students and increase their confidence in adopting emerging technologies [9, 10, 11, 12].

In today's technology‐driven society, gender differences in technology use and attitudes have been widely discussed in educational and psychological research [13, 14]. Historically, female students have been perceived as showing less interest in technology and holding more negative attitudes, contributing to the so‐called ‘technological gender gap’ [15, 16]. However, as digital technologies, including those used in healthcare and dentistry, become more present in everyday life and education, these attitudes are likely to shift [17]. While recent studies have explored gender‐related differences in attitudes toward technology, findings remain inconsistent. Some report more positive attitudes among male students, while others find no significant gender differences [18, 19]. In the context of dental education, it is important to explore whether such trends extend to students' perceptions and acceptance of digital tools like CAD/CAM systems.

In addition to formal education, students' attitudes and familiarity with digital tools can be influenced by informal factors, such as family background. Students with close relatives working in dentistry may have more opportunities to observe clinical practice, access technical information and receive encouragement to engage with advanced technologies. A study by Kettler et al. [20] demonstrated that early exposure to the dental profession can significantly affect students' career interests and enhance their adaptability to technological developments in dentistry.

Although numerous studies have investigated dental students' perceptions and competencies related to CAD/CAM technology in various international settings, there is a noticeable lack of empirical data from Croatia. This study aims to fill that gap by providing the first data‐driven insights into Croatian dental students' knowledge, attitudes and experience with CAD/CAM systems. Understanding how students engage with these technologies in both preclinical and clinical settings allows educators to identify deficiencies and guide curricular reform. As CAD/CAM becomes an increasingly essential part of modern prosthodontics, such localized assessments are critical to ensuring national programs remain globally competitive.

The objective of this cross‐sectional study was to assess dental students' awareness, attitudes and self‐reported technical knowledge of CAD/CAM technology, including their familiarity with specific clinical applications such as milling time and material properties, at the Faculty of Dental Medicine and Health in Osijek, Croatia. The study also examined whether year of study, gender, or family background in dentistry influenced students' perceptions and understanding of digital dental technologies. These findings aim to inform targeted curriculum development and promote more effective integration of CAD/CAM systems into undergraduate education.

## 2 Materials and Methods

### 2.1 Study Design and Setting

This cross‐sectional study was conducted at the Faculty of Dental Medicine and Health, Josip Juraj Strossmayer University of Osijek. Ethical approval was obtained from the Ethics Committee of the Faculty (Approval No: EP‐2024/25‐017). Participation was entirely voluntary, anonymous and based on informed consent.

### 2.2 Participants

The study included students enrolled in the 3rd, 4th, 5th and 6th years of the integrated undergraduate dental medicine program. A total of 102 students participated, of whom 44 (44.88%) were in preclinical training (3rd and 4th years) and 58 (55.12%) in clinical training (5th and 6th years). A power analysis indicated that a sample size of 64 participants would be sufficient to detect a medium effect size (Cohen's *d* = 0.5) with a significance level of 0.05 and statistical power of 80%. Therefore, the final sample of 102 students provided adequate power for the planned statistical analysis. The only exclusion criterion was incomplete responses. Demographic data collected included age, gender, year of study and whether the student had close family members working in the dental profession. The sample comprised 61 female and 41 male students.

### 2.3 Instrument

Data were collected using a self‐administered online questionnaire developed specifically for this study (File S1). The questionnaire was distributed via Google Forms and consisted of 21 items divided into two main sections:

- Section 1 (Demographics): Four questions covering gender, age, year of study (preclinical or clinical) and family background in dentistry
- Section 2: Seventeen questions assessing students' awareness, theoretical and practical knowledge, opinions regarding clinical applications and interest in further education related to CAD/CAM technology.

The questions included:

1. Have you heard of CAD/CAM?
2. Where did you hear about CAD/CAM?
3. Have you ever seen any CAD/CAM restoration?
4. Have you ever worked with CAD/CAM?
5. Have you ever used an intraoral scanner?
6. Did you receive any theoretical or practical CAD/CAM training in dental school?
7. Have you attended any CAD/CAM course outside the regular curriculum?
8. Should CAD/CAM be more thoroughly taught at the Faculty?
9. Do you consider CAD/CAM faster, too complex, or not cost‐effective compared to conventional techniques?
10. Do you feel sufficiently informed about CAD/CAM?
11. How much time is needed to mill a single crown?
12. Do zirconia oxide blocks have a shrinkage factor?
13. Which CAD/CAM system would you prefer to use?
14. Do you plan to pursue further education in CAD/CAM?
15. Do you believe that investing in CAD/CAM education is worthwhile?
16. What is your overall knowledge about CAD/CAM?
17. What is your overall opinion about CAD/CAM in dentistry?

The questionnaire was adapted from a previously validated instrument [21], with minor modifications to align with the Croatian dental education context. The original English version was translated into Croatian following established guidelines [22]. Translation was carried out jointly by a professional translator familiar with dental terminology and a bilingual dentist. The translated version was reviewed independently by two Croatian dentists and one dental student fluent in English. It was then back‐translated by another translator and reviewed by a native English speaker for consistency with the original version. Before back‐translation, a pilot study involving 15 students was conducted to assess question clarity. Psychometric evaluation included content validity, confirmed by an expert panel. A second pilot test with 30 students confirmed the clarity and relevance of the items. Internal consistency was measured using Cronbach's alpha, which yielded a value of 0.89, indicating acceptable reliability.

### 2.4 Questionnaire Structure

The questionnaire consisted of closed‐ended questions (Yes/No, not sure and multiple‐choice) and Likert‐type items. Students' self‐assessed overall knowledge of CAD/CAM technology was measured using a 10‐point Likert scale (1 = no knowledge, 10 = complete knowledge). Overall opinion of CAD/CAM technology was assessed using a 5‐point scale (0 = very negative, 4 = very positive).

### 2.5 Data Analysis

Survey responses were exported to Microsoft Excel and analysed using IBM SPSS Statistics version 26. Descriptive statistics were used to summarise demographic characteristics and questionnaire responses, including means (M), standard deviations (SD) and ranges where applicable. Normality of continuous variables was assessed using the Shapiro–Wilk test. Parametric tests (one‐way ANOVA and independent samples *t*‐tests) were used where assumptions of normality were met; otherwise, non‐parametric alternatives were considered. Chi‐square (*χ* ^2^) tests were used to examine associations between categorical variables. A significance level of *p* < 0.05 was applied to all statistical tests.

## 3 Results

A total of 102 participants were included in the study and divided into two groups: 44 students (44.88%) in preclinical training (PT) and 58 students (55.12%) in clinical training (CT). The overall average age was 24.38 years (SD = 2.06), with a range of 21–37 years. PT students were aged between 21 and 25 years (mean = 23), while CT students ranged from 21 to 37 years (mean = 25). Of all participants, 41 (40.20%) were male and 61 (59.80%) were female. Approximately half of the students (49.02%) reported having at least one family member employed in the dental profession.

Descriptive analysis indicated that students demonstrated a moderate level of knowledge about CAD/CAM technology, with a mean score of 5.49 out of 10. Their overall opinion of CAD/CAM was generally positive, with an average score of 3.38 out of 4 (SD = 1.22). The combined score for knowledge and attitude was 8.87 out of 14 (SD = 4.16) (Table 1).

**TABLE 1:** Descriptive statistics of the scale and subscales assessing students' knowledge and opinions on CAD/CAM technology.

Question (scale)	Mean ± SD	*N*	Min	Max
Q16. Knowledge of CAD/CAM technology	5.49 ± 3.25	102	1	10
Q17. Opinion on CAD/CAM technology	3.38 ± 1.22	102	0	4
Total score (knowledge + opinion)	8.87 ± 4.16	102	1	14

Abbreviations: Max, maximum; Min, minimum; *N*, number of participants; SD, standard deviation.

Detailed questionnaire results are presented in Tables 2, 3, 4, 5. A large majority of students (88.24%) reported having heard of CAD/CAM technology, but only 45.10% had any practical experience with it. Furthermore, 88.24% had never attended CAD/CAM‐related courses outside of the regular curriculum. Despite this, 90.20% believed CAD/CAM should be more thoroughly integrated into the curriculum, and 85.29% expressed interest in pursuing further education in this area (Table 2). Only 29.41% of students felt sufficiently informed about CAD/CAM. Notably, 54.5% of preclinical students and 32.75% of clinical students indicated that they did not feel sufficiently informed (*p* < 0.05). Additionally, 57.84% of students had used an intraoral scanner, and 85.29% considered investment in CAD/CAM education worthwhile (Table 2).

**TABLE 2:** Student experience and education in CAD/CAM technology.

Question	Yes (%)	No (%)	*N*
Q1. Have you heard of CAD/CAM?	80.39	19.61	102
Q3. Have you ever seen any CAD/CAM restoration?	55.88	44.12	102
Q4. Have you ever worked with CAD/CAM?	45.10	54.90	102
Q5. Have you ever use an intraoral scanner?	57.84	42.16	102
Q6. Did you receive any theoretical or practical CAD‐CAM training in dental school?	45.57	56.43	102
Q7. Have you attend any CAD/CAM course (outside the regular curriculum at the Faculty)	11.76	88.24	102
Q8. Should CAD/CAM be more thoroughly taught at the Faculty?	90.20	9.80	102
Q10. Do you feel sufficiently informed about CAD/CAM?	29.41	70.59	102
Q14. Do you plan to pursue further education in CAD/CAM?	85.29	14.71	102
Q15. Do you believe that investing in CAD/CAM education is worthwhile?	85.29	14.71	102

Abbreviation: *N*, number of participants.

Regarding system preferences, the majority of students (55.88%) favoured the InOffice CAD/CAM system, while 28.43% preferred the InLab system (Figure 1). In terms of perceived efficiency, 77.45% of students considered CAD/CAM technology significantly faster than conventional techniques. Only a small proportion viewed the technology as too complex (4.90%) or not cost‐effective (7.84%) (Figure 2). Concerning technical knowledge, 48.04% of respondents believed that milling a single crown takes < 1 h, whereas 45.10% were unsure. Furthermore, only 37.25% correctly identified that zirconia oxide blocks have a shrinkage factor. These results are summarised in Figure 3.

**FIGURE 1:**
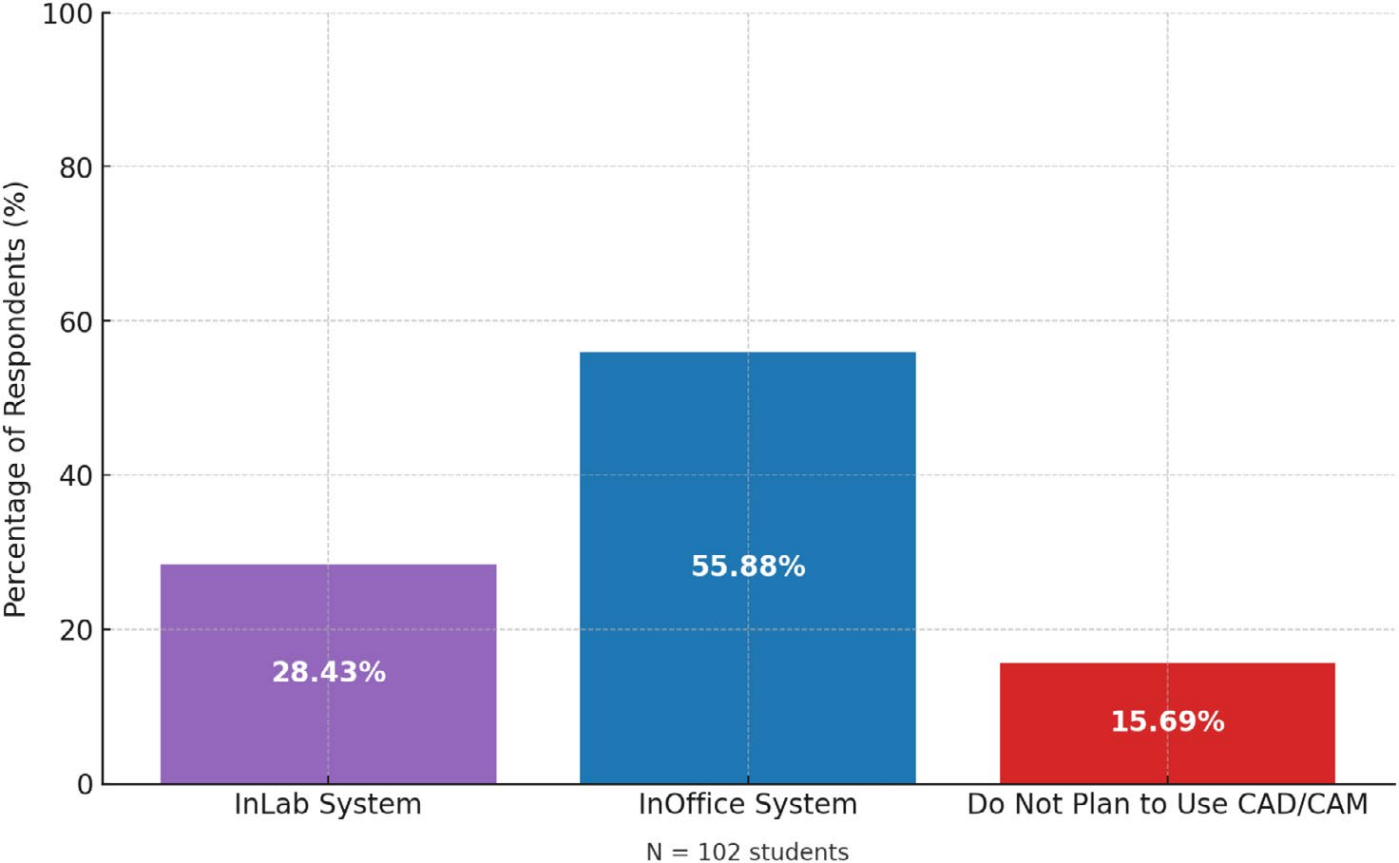
Preferred type of CAD/CAM system among students (Q13).

**FIGURE 2:**
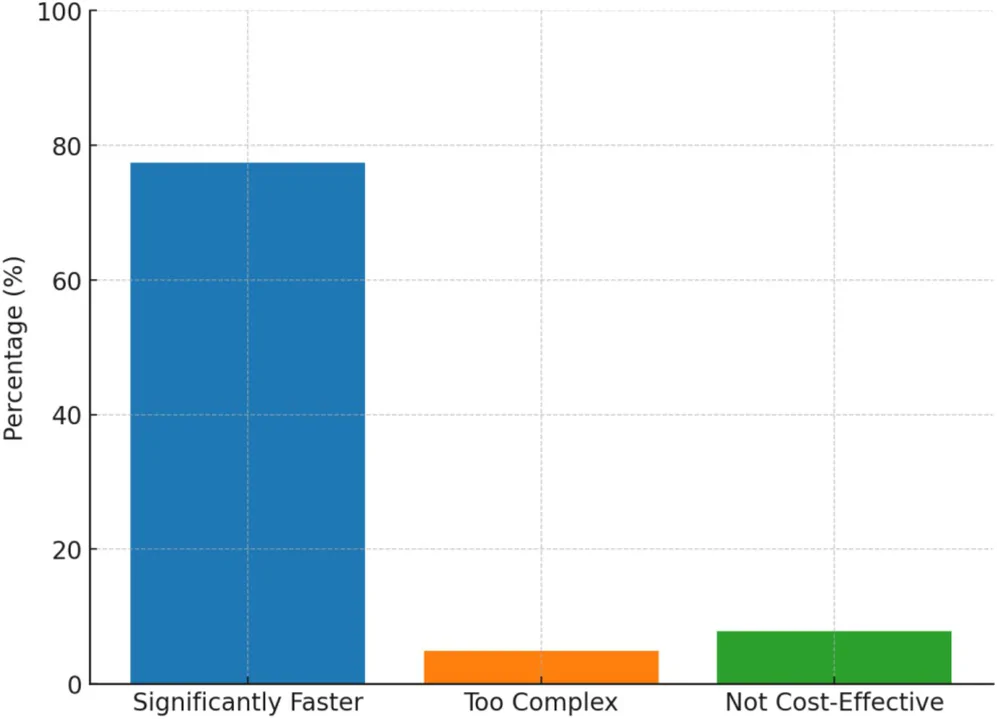
Students' perceptions of CAD/CAM technology compared to conventional techniques (Q9).

**FIGURE 3:**
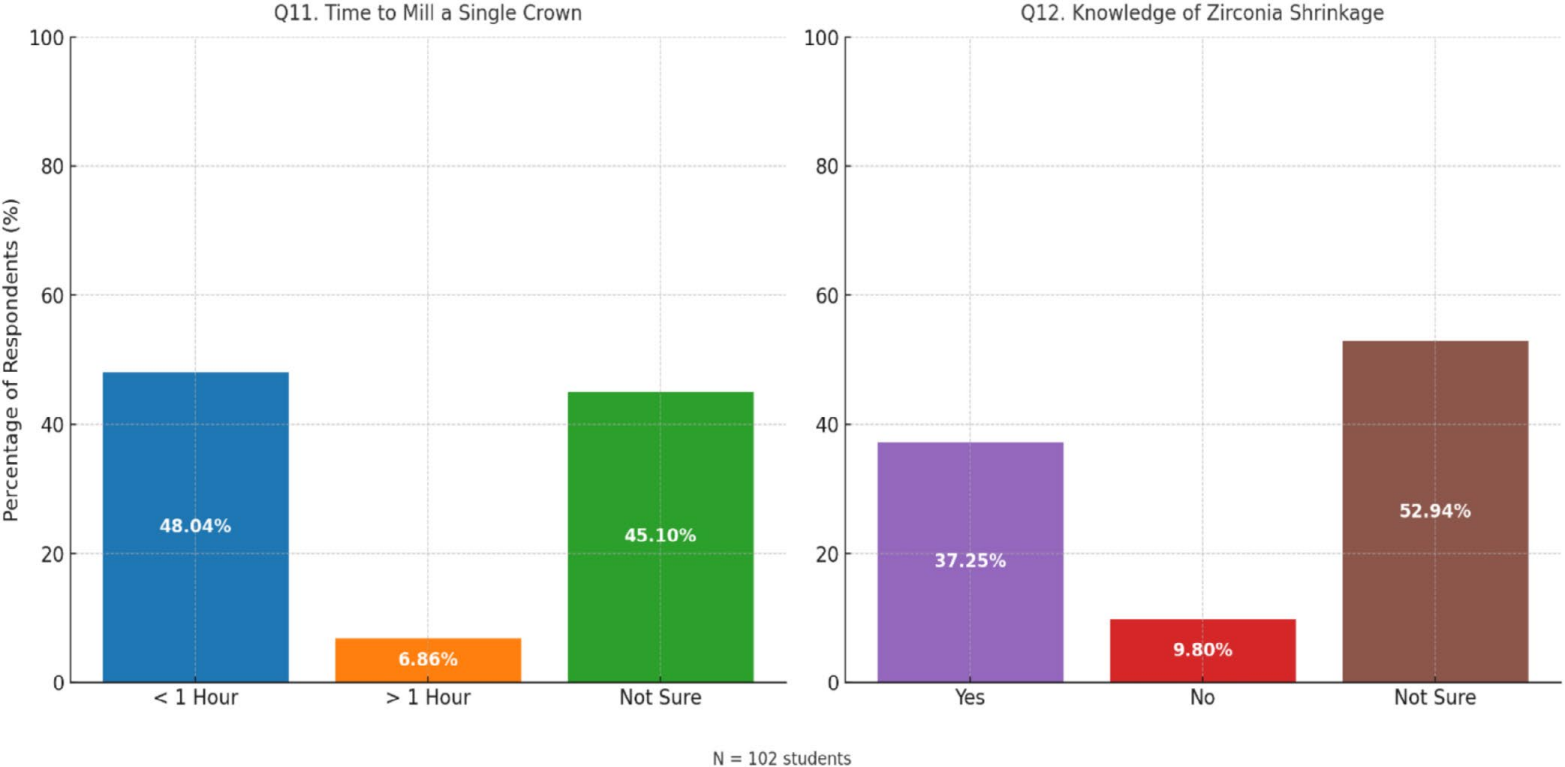
Student responses to technical aspects of CAD/CAM technology.

Statistical analysis revealed a significant difference in knowledge and attitudes across year levels (*F* = 14.79, *p* < 0.001), with higher scores among 5th‐ and 6th‐year students. Post hoc testing confirmed that CT students (5th and 6th year) had significantly greater knowledge and more positive attitudes toward CAD/CAM technology than PT students (3rd and 4th year) (*p* < 0.05) (Table 3).

**TABLE 3:** Knowledge and attitudes toward CAD/CAM technology among students by year of study.

Year of study	Mean ± SD	*N*	*F*	*p*
3rd year	7.13 ± 4.54	23		
4th year	5.52 ± 3.74	21		
5th year	9.96 ± 3.01	25	14.79	< 0.001*
6th year	11.39 ± 2.82	33		

*Note:* The * denotes statistical significance at the 0.05 level (*p*<0.05).

Abbreviations: *F*, *F*‐ratio; *N*, number of participants; *p*, significance level; SD, standard deviation.

A *t*‐test for independent samples was used to test the difference in students' knowledge and opinions about the use of CAD/CAM technology based on gender. The results showed that there was no statistically significant difference in knowledge or attitudes between male and female students (*t*(100) = 1.23, *p* = 0.222) (Table 4).

**TABLE 4:** Knowledge and attitudes toward CAD/CAM technology by gender.

Gender	Mean ± SD	*N*	*t*	*p*
Male	9.49 ± 3.96	41		
Female	8.46 ± 4.27	61	1.23	0.222

Abbreviations: *N*, number of participants; *p*, significance level; SD, standard deviation; *t*, *t*‐ratio.

However, a significant difference was observed between students with family members in the dental profession and those without such a background. Students with relatives in dentistry showed significantly higher knowledge and more positive attitudes toward CAD/CAM technology (*t*(100) = 4.12, *p* < 0.001) (Table 5).

**TABLE 5:** Knowledge and attitudes toward CAD/CAM technology based on family background in dentistry.

Family background	Mean ± SD	*N*	*t*	*p*
Yes	10.48 ± 3.27	50		
No	7.33 ± 4.36	52	4.12	< 0.001*

*Note:* The * denotes statistical significance at the 0.05 level (*p*<0.05).

Abbreviations: *N*, number of participants; *p*, significance level; SD, standard deviation; *t*, *t*‐ratio.

## 4 Discussion

Digital technologies have become an integral part of modern dental practice, transforming diagnostic procedures, treatment planning and the fabrication of prosthetic restorations. As digitalization continues to reshape the dental profession, it is essential that dental education evolves in parallel to adequately prepare future practitioners [6, 23]. Today's dental students belong to a generation that regularly interacts with technology, which shapes their learning preferences and expectations. Today's dental students belong to a generation that regularly interacts with technology, which shapes their learning preferences and expectations. As a result, there is growing demand for the integration of digital tools, such as CAD/CAM systems, into both theoretical and clinical dental training. This shift presents a challenge for educators, who must continuously adapt their teaching methods and update curriculum to reflect technological advancements.

As early as 2010, the importance of incorporating digital technologies into dental education was acknowledged [24]. Recognising the need for digital competencies, numerous dental schools worldwide have begun incorporating CAD/CAM technology into undergraduate programs to better equip students for contemporary clinical practice [10, 25].

Although a substantial body of literature exists on the clinical applications of CAD/CAM technology, relatively few studies have focused on dental students' knowledge, attitudes and awareness of these tools [7, 22, 23, 26]. This gap is particularly evident in the context of Croatian dental education, where empirical data are limited.

The present study provides valuable insight into the level of knowledge, perceptions and experiences of dental students at the Faculty of Dental Medicine and Health in Osijek regarding CAD/CAM technology. The findings confirm that although a high percentage of students are theoretically familiar with CAD/CAM, there remains a significant gap in practical exposure and hands‐on experience. While Hall et al. reported that nearly three‐quarters of students in their study had received CAD/CAM training during dental school and had high levels of practical exposure, our results show that most students lacked such experience, despite expressing strong theoretical awareness and positive attitudes [21]. Many participants had never seen a CAD/CAM restoration or worked with CAD/CAM equipment. These findings are consistent with previous studies [27, 28]. Most students learned about CAD/CAM technology through formal faculty coursework but had not attended additional courses outside the official faculty curriculum.

Furthermore, 90% of students agreed that CAD/CAM should be taught more comprehensively within the dental curriculum and expressed a strong desire for further training. These results align with findings from other studies, which indicate that incorporating CAD/CAM technology into both preclinical and clinical teaching positively influences students' attitudes and acceptance of digital tools [10, 29]. In contrast, Tran et al. reported that dental practitioners in the UK are rarely introduced to CAD/CAM during formal dental education. Instead, they often learn about it through external courses, company‐sponsored training, or by learning from colleagues [30]. Similarly, Hall et al. found that students from private institutions and those who gained exposure through workshops or social media had significantly higher KAP scores, highlighting the importance of diverse educational formats [21].

The current study also confirmed that students consider CAD/CAM technology valuable, particularly for its efficiency in producing prosthetic restorations compared to conventional methods. Only a small percentage of students viewed CAD/CAM as overly complex or not cost‐effective, which is consistent with Maltar et al.'s findings [28].

In terms of technical knowledge, nearly half of respondents believed that milling a single crown takes < 1 h, while 45.10% were uncertain. Only 37.25% correctly identified that zirconia oxide blocks undergo shrinkage during sintering. For comparison, only 11.4% of students in Kavarthapu and Suresh's study [31] and 20.7% in Maltar et al.'s study [28] answered this question correctly. In terms of system preference, most students favoured the InOffice CAD/CAM system, while 28.43% preferred the InLab system. This gap in mechanical knowledge likely results from limited opportunities to translate theory into practice, as CAD/CAM is currently taught primarily through lectures, without practical, hands‐on experience during undergraduate training.

Notably, senior students (5th and 6th year) exhibited significantly higher levels of knowledge and more positive attitudes toward CAD/CAM than their junior counterparts (3rd and 4th year). These results are consistent with Maltar et al., who found that final‐year students were more frequently involved in the clinical application of CAD/CAM technologies [28]. Similar trends were observed at the Faculty of Dentistry in Saudi Arabia, where clinical‐year students demonstrated greater knowledge than preclinical students [3]. Likewise, Islam et al., in a study conducted across the MENA region, reported that only about half of dental institutions offer CAD/CAM training at both the preclinical and clinical levels [32]. Our findings reflect similar patterns, emphasising the importance of structured curricular integration to gradually develop students' digital competence. Consistent with other regions, our participants strongly supported incorporating CAD/CAM training more deeply into undergraduate education, highlighting a widespread demand for curricular reform.

These findings underscore the need for a curriculum that provides structured, hands‐on exposure to CAD/CAM technology across both preclinical and clinical stages. Based on our data and findings from international literature, we recommend implementing a formal CAD/CAM module in the undergraduate dental curriculum that combines theoretical lectures, digital simulations and supervised clinical practice. Such a module would ensure that students not only understand the principles of digital dentistry but also develop the competence required for real‐world clinical application upon graduation.

In examining gender‐based differences, our study found no statistically significant variation in students' knowledge or attitudes toward CAD/CAM technology. This finding is supported by Hwisa et al., who reported that while males more often mentioned crown and bridge fabrication and females more often cited smile design and inlays/onlays, both genders demonstrated comparable overall familiarity [33]. These differences may reflect curriculum structure or cultural influences. Nonetheless, our findings emphasize the importance of ensuring equal access to digital education for all students, regardless of gender, and fostering an inclusive learning environment that encourages competency development in emerging technologies.

Gender differences in technology usage continue to be a topic of academic and societal interest [34, 35]. Historically, females have been perceived as having more negative attitudes toward technology and being less involved in technical fields, contributing to the so‐called ‘technological gender gap’ [15]. However, the widespread adoption of technology among younger generations appears to be diminishing these disparities [17]. Recent studies exploring gender‐related attitudes toward digital tools have produced mixed results. For example, Sáinz and López‐Sáez [18] reported that boys had more positive attitudes toward computers, whereas a later study by Sáinz et al. [19] found no significant gender differences. Our study aligns with the latter, showing that both male and female students demonstrated comparable knowledge and attitudes toward CAD/CAM.

This finding is particularly relevant, as it may reflect equitable access to educational content, learning resources and technological opportunities—especially within the context of egalitarian European societies such as Croatia. Although many existing studies support the idea of gender‐neutral attitudes toward CAD/CAM technology [36, 37, 38], it is important to recognize that these findings contrast with reports from regions where gender disparities in access to education and digital tools are more pronounced [39]. This contrast underscores the inclusive nature of our academic environment, where both male and female students appear equally empowered to engage with and advance in emerging digital competencies such as CAD/CAM.

In addition, students with family members working in the dental profession demonstrated significantly higher levels of knowledge and more positive attitudes toward CAD/CAM technology. This suggests that familial influence and early exposure to the dental field may play an important role in shaping students' perceptions of and engagement with modern technologies. It is likely that students with parents or close relatives in dentistry have greater access to technical information, informal mentoring and motivational support, which together foster a more proactive approach to learning and technology adoption. Each instance of exposure, whether through observation, discussion, or hands‐on practice, may contribute meaningfully to students' ability to effectively apply digital tools in clinical settings.

Similar observations have been made in prior studies, which found that students with family ties to the dental profession tend to exhibit greater initial awareness of technological trends and are more influenced and motivated in their academic and career decisions [40, 41]. This early exposure likely enhances their adaptability and enthusiasm for integrating innovations such as CAD/CAM into both their education and future clinical practice.

## 5 Limitations

This study has several limitations. First, it was conducted at a single institution with a relatively small sample size, which may limit the generalizability of the findings. Second, the use of self‐reported questionnaires introduces potential sources of bias, such as social desirability and inaccuracies in self‐assessment, which may obscure the actual level of exposure to CAD/CAM technology. Future research should include larger, multi‐institutional samples to improve representativeness. Moreover, employing mixed‐methods approaches that combine quantitative and qualitative data could provide deeper insights into students' experiences and attitudes. It would also be beneficial to investigate the impact of various educational formats, such as workshops and hands‐on training, on students' knowledge and attitudes toward CAD/CAM technology. Longitudinal studies tracking students' development over time may also reveal how their familiarity and comfort with digital dental technologies evolve throughout their education.

## 6 Conclusion

This study presents the first empirical data from Croatia on dental students' engagement with CAD/CAM technology. While 88.24% of students reported being aware of CAD/CAM systems, only 45.10% had any hands‐on experience, and just 11.76% had attended extracurricular training related to CAD/CAM. Despite this limited exposure, 90.20% of students believed that CAD/CAM should be more thoroughly integrated into the dental curriculum, and 85.29% expressed interest in pursuing further education in this field. Students in clinical years and those with family members working in dentistry demonstrated significantly higher levels of knowledge and more positive attitudes, highlighting the impact of clinical exposure and informal learning on the development of digital competence.

To address the observed gaps, particularly in practical training, we strongly recommend the introduction of a structured CAD/CAM module within the undergraduate dental curriculum. This module should include comprehensive theoretical instruction, digital simulations and supervised clinical application, spanning both preclinical and clinical stages of education. Such an approach would ensure that graduates are not only knowledgeable about digital dentistry principles but also competent in the clinical use of CAD/CAM technologies, ultimately enhancing their readiness for modern dental practice.

## Funding

This research was funded by the project ‘Innovation of Therapeutic Procedures by Introducing New Materials and Methods in Surgical, Prosthetic and Implant‐Prosthetic Rehabilitation of Patients’ (project code INOVA‐Dent‐KPI), co‐financed by the National Recovery and Resilience Plan (NPOO) in Croatia.

## Conflicts of Interest

The authors declare no conflicts of interest.

## Supporting information


**Data S1:** eje70126‐sup‐0001‐Supinfo.docx.

## Data Availability

The data that support the findings of this study are available from the corresponding author upon reasonable request.
