# Supplementary material for: Knowledge, Attitudes and Practical Experience With CAD/CAM Technology Among Dental Students in Croatia: A Cross‐Sectional Study
Source: Eur J Dent Educ. 2026 Mar 3;30(4):2070–8. doi: 10.1111/eje.70126 (PMC13686657; doi:10.1111/eje.70126)
Supplement: Supplementary file 1 [file EJE-30-2070-s001.docx]

# Supplementary File 1.

# CAD/CAM Questionnaire

| Question | | Answers |
| --- | --- | --- |
| Demographic section | | |
| Q1) Gender | | Male |
|  |  | Female |
| Q2) Age | | ……. |
| Q3) Year of study | | 3rd year |
|  |  | 4th year |
|  | | 5th year |
|  | | 6th year |
| Q4) Family background in dentistry | | Yes |
|  | | No |
|  | | |
| Experience and Exposure to CAD/CAM technology,  Knowledge-related questions,  Attitudes and future expectations | | |
| Q1) Have you heard of CAD/CAM? | | Yes |
|  |  | No |
| Q2) Where did you hear about CAD/CAM?  (multiple answers allowed) | | Conferences/webinars |
|  | | At the Faculty |
|  | | Hands-on workshops/courses outside the dental school |
|  | | Social media |
|  | | Books/articles |
|  | | Colleagues |
| Q3) Have you ever seen any CAD/CAM restoration? | | Yes  No |
| Q4) Have you ever worked with CAD/CAM? | | Yes |
|  | | No |
| Q5) Have you ever use an intraoral scanner? | | Yes |
|  | | No |
| Q6) Did you receive any theoretical or practical CAD-CAM training in dental school? | | Yes |
|  | | No |
| Q7) Have you attend any CAD/CAM course (outside the regular curriculum at the Faculty)? | | Yes |
|  | | No |
| Q8) Should CAD/CAM be more thoroughly taught at the Faculty? | | Yes |
|  | | No |
| Q9) Do you consider CAD/CAM technology faster, too complex, or not cost-effective compared to conventional techniques? | | Sigificantly faster  Too complex  Not cost-effective |
| Q10) Do you feel sufficiently informed about CAD/CAM? | | Yes |
|  | | No |
| Q11) How much time is needed to mill a single crown? | | < 1 Hour |
|  | | > 1 Hour |
|  | | Not sure |
| Q12) Do zirconia oxide blocks have a shrinkage factor? | | Yes |
|  | | No |
|  | | Not sure |
| Q13) Which type of CAD/CAM system would you prefer to use? | | InLab System |
|  | | InOffice System |
|  | | Do not plan to Use CAD/CAM |
| Q14)Do you plan to pursue further education in CAD/CAM? \| | | Yes |
|  | | No |
| Q15) Do you believe that investing in CAD/CAM education is worthwhile? | | Yes |
|  | | No |
| Q16) What is your overall knowledge about CAD/CAM? (1 =complete lack of knowledge or total disagreement, and 10 represented complete knowledge or full agreement) | | 10- Excellent |
|  | | 9- Very good |
|  | | 8- Good |
|  | | 7 -Above Average |
|  | | 6 -Average |
|  | | 5 -Acceptable |
|  | | 4 -Below Average |
|  | | 3 – Fair |
|  | | 2-Poor |
|  | | 1-Very Poor |
| Q17) What is your overall opinion about CAD/CAM in dentistry? | | 4-very postitive |
|  | | 3-positive |
|  | | 2- neutral |
|  | | 1-negative |
|  | 0-very negative | |
